# Structural and mechanistic basis of ADAR1-mediated RNA editing and immune regulation

**DOI:** 10.1016/j.cellin.2026.100299

**Published:** 2026-01-24

**Authors:** Xiangyu Deng, Mariam Elsharkawy, Yang Gao

**Affiliations:** a Department of Biosciences, Rice University, Houston, TX, 77005, United States; b Center for Nanoscale Imaging Sciences, Rice University, Houston, TX, 77005, United States

**Keywords:** ADAR1, RNA editing, dsRNA recognition, Innate immune regulation

## Abstract

Adenosine deaminase acting on RNA 1 (ADAR1) is a central regulator of innate immunity. By binding to and catalyzing adenosine-to-inosine deamination within double-stranded RNAs, ADAR1 mitigates the immunogenicity of self-derived RNAs and preserves cellular homeostasis. In this review, we summarize recent structural and mechanistic advances that illuminate key features of ADAR1 architecture, including how its multi-domains engage dsRNA substrates and contribute to substrate selectivity. Integrated with decades of biochemical and genetic studies, these insights refine our understanding of ADAR1's catalytic mechanism, domain-specific activities, and roles in suppressing immune signaling. We further highlight emerging knowledge on ADAR1's RNA substrate landscape, its interactions with protein partners, and the mechanistic principles that underlie its broad RNA editing and immune regulatory functions, with implications for disease pathogenesis and therapeutic RNA editing.

## 1 Introduction

The innate immune system relies on pathogen-associated molecular patterns (PAMPs) to distinguish self from non-self molecules and initiate host defense responses (Akira et al., 2006; Gordon, 2002). Double-stranded RNA (dsRNA) molecules, which represent replication intermediates produced during virus infections, serve as critical PAMPs that activate various RNA sensors to trigger interferon responses (Hur, 2019). However, endogenous RNAs can also form dsRNA structures, which, if misrecognized as non-self, may aberrantly activate immune sensors and drive autoimmune disorders (Chen and Hur, 2022; Reich and Bass, 2019). Thus, active mechanisms are required to discriminate self-derived dsRNAs from pathogenic counterparts to prevent harmful hyperactivation of innate immunity. Adenosine deaminase acting on RNA 1 (ADAR1) is a key immune surveillance factor of dsRNA (Bass, 2002; Kim et al., 1994). Supporting its essential function, ADAR1 knockout in mice results in embryonic lethality due to unchecked interferon responses (Hartner et al., 2004; Wang et al., 2004). In humans, ADAR1 mutations cause Aicardi–Goutières syndrome (AGS), a severe interferonopathy marked by constitutive interferon signaling (Nakahama et al., 2021; Rice et al., 2012; Tang et al., 2021).

Adenosine deamination by ADAR enzymes converts adenosine (A) to inosine (I), which base-pairs preferentially with cytidine (C) rather than uridine (U). Because cellular machinery interprets inosine as guanosine (G), A-to-I editing manifests as A-to-G changes that can alter RNA coding potential in translation, splicing, and silencing (Bass, 2002; Kawahara et al., 2007; Rueter et al., 1999; Tang et al., 2020). The unique ability of ADAR1 for programmable RNA recoding has attracted extensive interest in harnessing it for therapeutic RNA editing. Guide RNA-directed editing tools leveraging endogenous ADAR1 offer a promising platform for precise and programmable correction of disease-associated mutations (Bertoli et al., 2025; Booth et al., 2023; Katrekar et al., 2019; Merkle et al., 2019; Qu et al., 2019; Sun et al., 2025). While in dsRNA, I:U pairs resemble G:U wobble pairs but are more dynamic and undergo increased base-pair opening (Müller-Hermes et al., 2025). When editing occurs at multiple adjacent sites, the cumulative destabilization can weaken or remodel local RNA secondary structures (Bass, 2002; Reich and Bass, 2019). Indeed, ADAR1 was initially hypothesized to function as an RNA helicase because extensive editing can produce an apparent “unwinding” effect (Bass and Weintraub, 1988; Wagner et al., 1989).

Through binding to and editing of diverse dsRNAs, ADAR1 can efficiently reduce the immunogenicity of endogenous dsRNAs, thereby restraining aberrant immune activation (Li and Walkley, 2025; Quin et al., 2021). Genetic studies suggest that ADAR1 plays a central role in regulating RNA sensors MDA5, PKR, and ZBP1: lethality of ADAR1 knockout can be rescued by combined knockout of MDA5 and PKR, and mutations affecting ADAR1 Z-RNA binding induce ZBP1-mediated cell death(de Reuver et al., 2022; Hu et al., 2023; Hubbard et al., 2022; Li et al., 2022; Liddicoat et al., 2015; Mannion et al., 2014; Sun et al., 2025; Zhang et al., 2022). Notably, these immune sensors recognize distinct RNA substrates, suggesting that ADAR1 must engage and edit diverse endogenous dsRNAs to antagonize multiple pathways. These observations highlight several unresolved questions: What are the endogenous ADAR1 substrates for different immune sensors’ regulation? How does ADAR1 use its multiple domains to bind and edit these dsRNAs for immune regulation? How do ADAR1 interaction partners modulate ADAR1 editing and other RNA metabolic pathways? In this review, we will focus on the structural features of ADAR1 and emerging insights into its RNA substrates and interacting proteins, while referring readers to other recent excellent reviews for in-depth discussions of genetics, physiological, and pathological roles of ADAR1 (Baker and Slack, 2022; de Reuver and Maelfait, 2024; Heraud-Farlow and Walkley, 2020; Li and Walkley, 2025; Marônek et al., 2025).

## 2 Structure and mechanism of ADAR1

ADAR enzymes share a conserved domain organization, consisting of a number of N-terminal RNA-binding modules and a C-terminal Zn^2+^-dependent deaminase domain (Bass, 2002). ADAR1 has the most complex architecture, containing two Z-RNA–binding domains (ZBDα and ZBDβ) followed by three double-stranded RNA-binding domains (RBDs 1–3) and a deaminase domain, and both a nuclear export signal (NES) and a nuclear localization signal (NLS). Human cells also express the shorter ADAR1 p110 isoform, which lacks ZBDα and NES, as well as two paralogs: ADAR2, which has two RBDs but no ZBDs or NES, and ADAR3, which contains two RBDs but an inactive deaminase domain and lacks NES (Bass, 2002). These isoforms differ in domain composition, subcellular localization, and tissue distribution. Among them, the interferon-inducible ADAR1 p150 isoform serves as the principal suppressor of aberrant innate immune activation, consistent with its expanded domain repertoire, cytoplasmic localization, and likely broader substrate promiscuity (Ahmad et al., 2018; Hartner et al., 2004; Li et al., 2022; Liddicoat et al., 2015; Quin et al., 2021; Sun et al., 2025; Tang et al., 2021). In this section, we summarize the unique structural features of ADAR1 in comparison with its paralogs to understand its role in immune regulation.

### 2.1 Deaminase domain

**Fig. 1:**
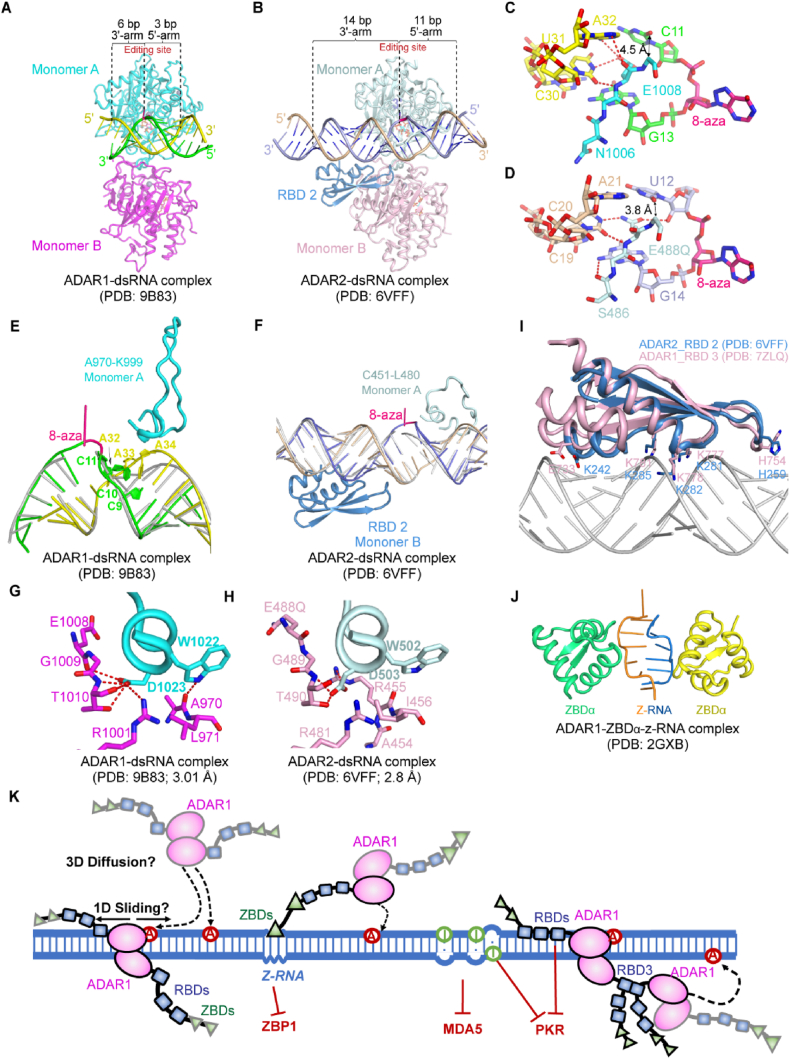
**Structural basis ofADAR1-mediatedRNA editing and immune regulation.** (A) Structure of the ADAR1–dsRNA complex (PDB: 9B83). The deaminase domain contacts a short (∼10 bp) dsRNA region surrounding the editing site. (B) Structure of the ADAR2–dsRNA complex (PDB: 6VFF). ADAR2 engages a substantially longer (∼25 bp) dsRNA footprint than ADAR1. RBD2 (blue) clamps the 3′ arm of the duplex. (C) Close-up view of the ADAR1 active site. The E1008 occupies the vacated space created by base flipping and interacts with the orphan base (U31). E1008 also engages the adenine on the non-editing strand (A32). (D) Close-up view of the ADAR2 active site. Residues E488Q and S486 make direct backbone-mediated contacts with the editing strand bases (U12 and G14), enforcing a constrained geometry at the editing site. Compared with ADAR1 (C), the distance between the U12 base and the E488Q Cα (∼3.8 Å) reflects tighter positioning that favors a canonical U–A base pair than a G-C base pair at the −1 position. (E) In the ADAR1–dsRNA complex, binding of the deaminase domain induces pronounced remodeling of the duplex relative to an ideal A-form helix (gray). Labeled AAA-CCC mismatches highlight locally distorted regions near the editing site, reflecting ADAR1 tolerance of mismatches. The RNA-binding loop (residues A970–K999) adopts an open conformation stabilized by an auxiliary Zn^2+^-binding site. (F) In the ADAR1–dsRNA complex, ADAR2 binding preserves a similar A-form RNA geometry (gray), relying on an extended RNA-binding interface formed by both the RNA-binding loop (residues C451–L480) and RBD2 to stably engage a well-paired duplex. (G) Dimerization interface of the ADAR1 deaminase domain. D1023 from Monomer A forms key stabilizing interactions that anchor the opposing catalytic loop in Monomer B. (H) Dimerization interface of the ADAR2 deaminase domain. D503 occupies an analogous position to D1023 in ADAR1 and mediates critical dimerization contacts. (I) Structural overlay of ADAR1 RBD3 (pink; PDB: 7ZLQ) and ADAR2 RBD2 (blue; PDB: 6VFF) bound to an A-form dsRNA, showing a conserved dsRBD fold and shared RNA-binding geometry. In both domains, conserved residues in helix α1, the β1–β2 loop, and helix α2 mediate minor-groove recognition through backbone contacts, including the invariant glutamate in α1, histidine in the β1–β2 loop, and the KKxxK motif in α2. (J) Crystal structure of the ADAR1 ZBDα bound to Z-RNA (PDB: 2GXB). ZBDα adopts a winged helix–turn–helix fold and recognizes the zig-zag backbone of left-handed Z-RNA. (K) Working model for ADAR1 action on endogenous dsRNA. ADAR1 is proposed to engage dsRNA substrates through multivalent interactions involving ZBDs and RBDs, followed by one-dimensional sliding and local sampling by the deaminase domain. Editing and competition for immunogenic dsRNA suppresses activation of cytoplasmic RNA sensors, including ZBP1, MDA5, and PKR, thereby maintaining immune homeostasis.

Structures of ADAR1 and ADAR2 deaminase domains in complex with dsRNA substrates have revealed a conserved architecture (Fig. 1(A) and (B)) (Deng et al., 2025; Thuy-Boun et al., 2020). All ADAR active sites contain a catalytic Zn^2+^ ion coordinated by the His–Glu–X_55_–PCX_65_C motifs, where the glutamate functions as the catalytic base for water deprotonation and subsequent hydrolytic deamination of adenosine (Fisher and Beal, 2024; Macbeth et al., 2005). In addition, ADAR1 and ADAR2 both bind a buried inositol hexakisphosphate (IP_6_) molecule that stabilizes the deaminase fold (Deng et al., 2025; Macbeth et al., 2005). A conserved Gly-Glu-Gly (GEG) motif within a flexible loop of the deaminase domain plays a central role in target adenosine recognition and editing. During RNA editing, the GEG motif penetrates the dsRNA minor groove and extrudes the target adenosine from the duplex, positioning it for hydrolytic deamination (Fig. 1(C) and (D)). The glutamate within the GEG motif (E488 in ADAR2; E1008 in ADAR1) fills the vacated space and pairs with the orphan base, giving them a preference for U or C opposite the edited adenosine (Deng et al., 2025; Matthews et al., 2016; Thuy-Boun et al., 2020). Although both enzymes frequently target sites within a 5′-UAG-3′ motif, their sequence-readout mechanisms differ. ADAR2 makes direct backbone contacts with the −1 U and +1 G near the GEG motif, and sequence deviations strongly reduce its activity (Fig. 1(D)) (Matthews et al., 2016; Thuy-Boun et al., 2020). In contrast, ADAR1 does not interrogate the −1 and + 1 positions as stringently. Its key residue, E1008 with its sidechain and backbone, also senses the +1 base, but does so on the non-editing strand for an “A” preference (Fig. 1(C)) (Deng et al., 2025). In addition, ADAR1 readily tolerates consecutive mismatches immediately 5′ of the edited adenosine, further contributing to its broader substrate permissiveness (Fig. 1(C)) (Deng et al., 2025). Although ADAR3 retains the conserved catalytic zinc center, essential residues for the base-flipping and deamination steps are missing or mispositioned, rendering it inactive (Chen et al., 2000).

ADAR1 deaminase domain contacts only a 10 bp RNA around the edited base, ∼3–4 bp 5′ arm of the adenosine, and a ∼6 bp 3′ arm of the adenosine (Fig. 1(A)) (Deng et al., 2025). In contrast, ADAR2 engages a substantially longer dsRNA segment, including ∼10–11 bp on the 5′ side and ∼7–8 bp (or 13–14 bp if the RBD2 is bound) on the 3′ side (Fig. 1(B)) (Matthews et al., 2016; Thuy-Boun et al., 2020). This difference arises in part from an alternative conformation of an RNA-binding loop, residues 970–999 in ADAR1 and residues 451–480 in ADAR2 (Fig. 1(E) and (F)). The loop contacts the 5′ side of dsRNA in ADAR2 (Fig. 1(F)), whereas in ADAR1, H988 within this loop forms an auxiliary Zn^2+^-binding site with C1081, C1082, and H1103, stabilizing an “open” conformation that limits direct RNA contacts (Fig. 1(E)) (Deng et al., 2025). Moreover, ADAR2 only induces constrained local adjustments to the helix during base flipping, including slight minor groove widening and shearing of flanking base pairs (Fig. 1(F)) (Matthews et al., 2016; Thuy-Boun et al., 2020). In contrast, ADAR1 remodels the 5′ arm of the duplex, introducing local distortions that disrupt hydrogen bonding between base pairs to help accommodate mismatches (Fig. 1(E)) (Deng et al., 2025). This structural flexibility allows ADAR1 to act on short non-ideal or mismatched RNAs and underlies its much broader sequence and structural tolerance compared with ADAR2.

Both ADAR1 and ADAR2 deaminase domains were found to form asymmetric homodimers, with one protomer inserting a conserved dimerization helix into the second protomer's catalytic surface (Fig. 1(G) and (H)) (Deng et al., 2025; Thuy-Boun et al., 2020). As a result, only one protomer is catalytically competent. In ADAR1, the second protomer additionally provides K1115 for dsRNA interaction, a feature absent in ADAR2. Mutation of the key D1023 within the ADAR1 dimerization helix abolishes editing (Deng et al., 2025). In ADAR2, mutation of the analogous residue D503 strongly disrupts dimerization but still permits activity on some RNAs, indicating substrate-dependent requirements for ADAR2 dimers (Thuy-Boun et al., 2020). The dimerization interface is highly conserved across ADARs (Fig. 1(G) and (H)), and ADAR1–ADAR2 heterodimers have been detected in cells, implying possible cooperative or competitive regulation (Anantharaman et al., 2017; Ashley et al., 2024; Thuy-Boun et al., 2020). AlphaFold2 predictions indicate that ADAR3 can also heterodimerize with ADAR1 and ADAR2 and always poises its dimerization loop across active sites of ADAR1 or ADAR2 for possible inhibitory regulation of editing (Fisher and Beal, 2024).

### 2.2 RBD domains

ADAR1 contains three RBDs, whereas ADAR2 and ADAR3 each contain two. Each RBD is ∼70 amino acids and adopts the canonical α1–β1–β2–β3–α2 fold, engaging one face of an A-form duplex across two adjacent minor grooves (Fig. 1(I)) (Rehwinkel and Mehdipour, 2025; Stefl et al., 2006). dsRNA recognition relies on three conserved features: a glutamate in α1 that contacts ribose 2′-OH groups, a histidine in the β1–β2 loop that probes the minor groove, and a KKxxK motif in α2 that binds the phosphate backbone (Ryter, 1998; Thomas and Beal, 2017). Early NMR structures suggested that ADAR2 RBDs might contribute to sequence readout through minor-groove interactions with specific bases (Stefl et al., 2010), while subsequent biochemical and structural analyses have not supported this model (Thomas and Beal, 2017).

The RBD adjacent to the deaminase domain, RBD3 in ADAR1 and RBD2 in ADAR2, plays an important role in deamination. Deleting or mutating RBD3 in ADAR1 severely reduces dsRNA binding and abolishes editing, confirming its essential role in substrate engagement (Deng et al., 2025). However, ADAR1 RBD3 has only been captured on the 3′ arm of the dsRNA in an inactive state, with the RBD3–deaminase linker occupying the active site. In catalytically competent structures of ADAR1, RBD3 appears disordered in the electron microscope map, possibly because of its non-specific RNA binding or significant flexibility (Deng et al., 2025). In contrast, ADAR2 RBD2 has been captured adjacent to the deaminase domain in active complexes, where it clamps the 3′ arm of the dsRNA through canonical dsRBD–minor-groove interactions (Fig. 1(F)) (Thuy-Boun et al., 2020). This architecture allows ADAR2 to bind tightly to long, uninterrupted A-form helices but may limit its ability to accommodate mismatches and structural irregularities. Notably, the ADAR2 deaminase domain retains activity without RBD2, in contrast to the strict RBD3 requirement observed for ADAR1 (Deng et al., 2025; Thuy-Boun et al., 2020). This distinction is further reflected in engineered RNA-editing systems with the CRISPR-Cas13 platform (Cox et al., 2017): the ADAR2 deaminase domain, when fused to Cas13, can efficiently engage dsRNA and support site-specific editing (Ishikawa et al., 2025); whereas fusion of the ADAR1 deaminase domain to Cas13 inhibits target editing by endogenous ADAR1, highlighting the strict dependence of ADAR1 on RBD3 for productive RNA engagement (Qu et al., 2019).

RBD1 and RBD2 may contribute to the basal RNA-binding in ADAR1, but how these domains shape enzyme activity and specificity remains unclear. Domain-swap analyses show that replacing ADAR1 RBD1 and RBD2 with their ADAR2 counterparts restricts editing to the canonical 5′-UAG-3′ motif, whereas introducing ADAR1 RBDs into ADAR2 broadens flanking-sequence tolerance and permits editing at non-canonical or structurally irregular sites (Rehwinkel and Mehdipour, 2025; Uzonyi et al., 2021; Zambrano-Mila et al., 2023). In addition, full-length ADAR2 is strongly autoinhibited on short duplexes, and truncation of RBD1 or RBD1 binding to long dsRNA relieves the inhibition (Macbeth et al., 2004). By contrast, deletion of RBD1 or RBD2 has only a minor effect on deamination of short hairpin substrates (Deng et al., 2025). This suggests that RBD1 and RBD2 may only facilitate recruitment of ADAR1 to long dsRNAs, but not directly contribute to substrate proofreading or catalysis.

### 2.3 ZBD domains

ADAR1 contains two ZBDs, ZBDα and ZBDβ, each adopting a winged–helix–turn–helix fold composed of three α-helices and a short β-sheet (Fig. 1(J)). Only ZBDα retains the ability to recognize the left-handed Z-RNA and Z-DNA, engaging the characteristic zig-zag phosphodiester backbone through residues in its α2–α3 region and β-wing (Krall et al., 2023; Schade et al., 1999; Schwartz et al., 1999). Moreover, helix α1 of ZBDα contains a leucine-rich NES, enabling ADAR1 p150 to shuttle between the nucleus and cytoplasm to suppress cytoplasmic immune sensors activation (Poulsen et al., 2001; Strehblow et al., 2002). Although ZBDβ shares the same overall topology, it has no Z-RNA binding activity due to substitutions of key basic and aromatic residues required for Z-conformation recognition (Kim et al., 2003; Schwartz et al., 1999). Recent analysis suggested that ZBDβ may instead interact with G-quadruplex RNA, hinting at a distinct regulatory role (Herbert et al., 2025; Kroft et al., 2025). Functionally, ZBDα is thought to recruit the cytoplasmic ADAR1 p150 isoform to transcripts capable of adopting Z-RNA structures, including Alu–Alu inverted repeats and other long repetitive dsRNAs (Herbert et al., 2025). The ZBDs may compete with ZBP1 for suppressing dsRNA-induced necroptosis, providing a mechanistic link between ADAR1 structural recognition and cell-death control (de Reuver et al., 2022; Hubbard et al., 2022; Jiao et al., 2022; Zhang et al., 2022).

## 3 ADAR1 editing of diverse RNA substrates

Deep RNA-sequencing analyses have shown that ADAR1 edits a broad spectrum of endogenous RNAs, including protein-coding and non-coding RNAs, repetitive Short and Long Interspersed Nuclear Elements (SINEs and LINEs), and long cis-natural antisense transcripts (cis-NATs) dsRNA generated by sense–antisense transcription (Brümmer et al., 2017; Li et al., 2009, 2018, 2022; Peng et al., 2012; Porath et al., 2014; Song et al., 2020; Tan et al., 2017). However, the *bona fide* immunogenic RNA substrate under ADAR1 regulation is still under debate (Levanon et al., 2024). Moreover, MDA5, PKR, and ZBP1 each recognize different features of dsRNA, and how ADAR1 effectively suppresses their activation remains unclear. Below, we summarize recent advances in defining ADAR1 substrates by structural classes and immune relevance, as well as the possible mechanisms of ADAR1 action.

### 3.1 dsRNA substrates for ADAR1 editing and innate immune activation

MDA5 is preferentially activated by long, uninterrupted dsRNAs, such as those produced during viral replication (Peisley et al., 2012). These extended duplexes provide a platform for MDA5 oligomerization, sliding, and cooperative filament assembly, which are required for productive signaling (Han et al., 2025). PKR activation is also RNA length dependent: while dsRNAs as short as ∼16 bp can bind PKR monomers, duplexes of ∼30 bp are required for kinase dimerization and activation, and longer dsRNAs (up to ∼100 bp) support optimal PKR activity (Ahmad et al., 2025; Lemaire et al., 2008; Zheng and Bevilacqua, 2004). In contrast, the mechanism of ZBP1 activation beyond its recognition of left-handed Z-RNA structures is less well defined but is thought to involve ligand-induced oligomer, filament, or phase separation formation (Devos et al., 2020; Kuriakose et al., 2016; Xie et al., 2024).

Short dsRNA structures are widespread in cellular RNAs, including mRNAs and structured noncoding RNAs such as pri- and pre-miRNAs (Li et al., 2009; Nishikura, 2016). In protein-coding transcripts, editing can introduce nonsynonymous changes, exemplified by the Gria2 (GluR-B) Q/R site that modulates AMPA receptor permeability (Sommer et al., 1991). In miRNAs, A-to-I editing within pri- or pre-miRNA hairpins can alter miRNA processing by Drosha or Dicer nucleases and miRNA targeting by Argonaute protein, thereby reshaping miRNA abundance and target specificity (Nishikura, 2016; Ota et al., 2013; Quinones-Valdez et al., 2019; Yang et al., 2006). Despite these potential molecular consequences, the short dsRNAs typically span only ∼20–100 bp and frequently contain bulges and mismatches, which likely limit their ability to support oligomerization and activation of RNA sensors. Furthermore, mice lacking ADAR enzymes engineered to carry pre-edited GluR-B Q/R sites develop normally (Chalk et al., 2019), suggesting that most short-dsRNA editing events are not essential under basal physiological conditions.

A substantial fraction of A-to-I editing in humans occurs within SINE Alu elements, which make up more than 10% of the genome (Levanon et al., 2004; Porath et al., 2014; Ramaswami and Li, 2014). Inverted Alu repeats embedded within transcripts frequently form imperfect dsRNAs of ∼200–500 bp containing mismatches, loops, and other noncanonical features. Alu-derived dsRNAs have been proposed as promising candidates for endogenous immunogenic ligands (Ahmad et al., 2018, 2025; Hundley and Bass, 2010; Morse et al., 2002). Gain-of-function AGS-associated MDA5 mutations can readily bind and be activated by Alu dsRNAs to induce interferon responses, phenocopying ADAR1 loss (Ahmad et al., 2018; Rice et al., 2014; Yu et al., 2021). PKR, which requires shorter and less perfectly paired dsRNAs for activation, has also been shown to respond readily to Alu RNAs (Ahmad et al., 2025; Chung et al., 2018). In addition, Alu elements may contain Z-RNA motifs that recruit ADAR1 and ZBP1 via its ZBDs, linking recognition of alternative RNA conformations to suppression of inappropriate immune activation (Nichols et al., 2021). Nevertheless, most Alu elements reside within introns and therefore do not reach the cytoplasm, limiting their accessibility to activate innate immune sensors. Moreover, because Alu duplexes are relatively short and structurally imperfect, they cannot robustly activate wild-type MDA5, the principal cytoplasmic dsRNA sensor (Ahmad et al., 2018; Yu et al., 2021).

Lastly, long, nearly perfect dsRNAs can form through bidirectional or antisense transcription, generating cis-NATs or overlapping intergenic duplexes (Lavorgna et al., 2004). These long A-form helices are potent MDA5 substrates, mimicking viral dsRNA. Recent evidence suggested that ADAR1 counteracts this by extensively editing such duplexes to prevent aberrant MDA5 activation (Hu et al., 2023; Sun et al., 2025). While editing has been documented at specific cis-NAT loci, the overall abundance of NAT-derived dsRNAs in mammalian cells remains unclear (Levanon et al., 2024). However, recent evidence suggested that stress conditions, such as disrupted transcriptional regulation, may induce long dsRNA formations that trigger immunogenic responses (Baluapuri et al., 2025).

### 3.2 Model of ADAR1 editing and immune suppression

To catalyze A-to-I editing, ADAR1 must first identify editable adenosines within a segment of dsRNA and flip it to the active site for deamination. Despite extensive biochemical and structural studies, the detailed mechanism by which ADAR1 locates and selects its target sites remains poorly understood. Many base-flipping enzymes locate target bases through a combination of one-dimensional (1D) sliding along nucleic acids and three-dimensional (3D) diffusion (Stivers and Jiang, 2003). Consistent with this model, recent single-molecule atomic force microscopy studies show that ADAR1 dimers can dynamically associate with and dissociate from dsRNA while also performing 1D sliding along the duplex (Fig. 1(K)) (Biyani et al., 2025). Sequence and structural features within dsRNA may further guide target recognition. The 5′-UAG-3′ motif could act as a preferred docking or recognition element, while mismatches or structural irregularities at or near the editing site may lower the energetic barrier for base flipping.

While such single or site-specific editing events could explain how ADAR1 engages dsRNA and catalyzes deamination, they are unlikely to fully account for ADAR1's physiological role in suppressing innate immune activation. Immune silencing likely requires substantial destabilization of long dsRNA substrates, a function depending on extensive and highly efficient hyperediting. Consistent with this idea, ∼70 editing sites have been identified within a 210-bp segment in the 5′ untranslated region of *Klf1*, a proposed physiological substrate of ADAR1 in mouse model (Liddicoat et al., 2015), and 62 editing sites were detected within a 500-bp region of cisNAT dsRNA composed of two overlapping genes, TNFRSF14 and TNFRSF14-AS1 (Li et al., 2022). However, the degree of editing required to sufficiently destabilize dsRNA and suppress activation of innate immune sensors remains unclear, as do the sequence and length features of dsRNAs that enable ADAR1 hyperediting. Notably, editing within long dsRNAs displays relatively relaxed site selectivity and can occur at adenosines embedded in imperfect sequence contexts, including within non-canonical A:A or A:G pairs, enabling widespread modification across the duplex (Athanasiadis et al., 2004; Levanon et al., 2004; Song et al., 2020). Mechanistically, such hyperediting may arise from the processive actions of one or a few ADAR1 molecules traversing long dsRNA, and/or from clustered or oligomeric assemblies of ADAR1 engaged on extended duplex substrates.

An ADAR1 dimer contains 6 RBDs and 4 ZBDs, providing extensive and multivalent RNA-binding capacity. These multiple RNA-binding domains may enable initial anchoring of ADAR1 to long or structured RNA duplexes, increasing local residence time and allowing the deaminase domains to efficiently sample nearby adenosines (Fig. 1(K)). Notably, the isolated deaminase domain is catalytically inactive, whereas an RBD3–deaminase construct retains substantial editing activity, underscoring a critical role for RBD3 in positioning or stabilizing the deaminase domain during catalysis (Deng et al., 2025). In contrast, the precise contributions of RBD1, RBD2, and the ZBDs to substrate engagement, particularly for hyperedited long dsRNA, remain unclear and likely vary depending on the length and local structural variations of the RNA substrate.

An “ADAR-recruiting element” has been proposed in the GluR-B Q/R stem loop and was incorporated into many guide-RNA designs to enhance recruitment of endogenous ADARs (Katrekar et al., 2022; Merkle et al., 2019; Reautschnig et al., 2022), yet long guide RNAs without this motif can still support robust editing (Katrekar et al., 2019; Sun et al., 2025; Yi et al., 2022). Similarly, while some Alu elements can form Z-RNA structures that are recognized by the ZBDs, recent studies indicate that Z-RNA contributes only modestly to overall editing efficiency (Nichols et al., 2025).

Several observations also suggest cooperative or oligomeric behavior during ADAR1-mediated hyperediting (Biyani et al., 2025; Song et al., 2020). Both the deaminase domain and RBD3 can homodimerize (Deng et al., 2025; Mboukou et al., 2024), and multivalent interactions between ADAR1 domains and long dsRNAs could promote ADAR1 clustering or even phase separation (Fig. 1(K)). Such assemblies could enhance local ADAR1 concentration and substrate engagement, accounting for the clustered hyperediting observed in long dsRNA. However, direct visualizations of higher-order ADAR1 assemblies or phase-separated condensates have not yet been achieved. Moreover, many editing sites are closely spaced along the duplex, which would sterically hinder the simultaneous binding of multiple ADAR1 molecules, arguing against a simple model of concurrent multimer occupancy at adjacent sites.

Hyperediting further introduces a mechanistic complication: progressive conversion of A:U pairs into I:U wobble pairs destabilizes the duplex and relaxes local helical structure (Nishikura, 2016). This relaxation may shift which adenosines remain accessible and thus alter ADAR1 processivity. ADAR1's intrinsic tolerance of mismatches and its capacity to act on short and imperfect duplex segments likely enables it to continue editing as the substrate becomes increasingly destabilized. Interestingly, AGS-associated mutations in ADAR1 selectively impair editing of short duplexes but not dsRNA substrates with long flanking regions *in vitro* (Deng et al., 2025). This defect may compromise hyperediting of long endogenous dsRNAs *in vivo*, leading to insufficient suppression of immunogenic RNA species and consequent aberrant interferon activation (Deng et al., 2025; Jiao et al., 2022; Nakahama et al., 2021; Rice et al., 2012). These observations highlight the importance of ADAR1's substrate promiscuity and its ability to hyperedit diverse duplexes for preventing aberrant activation of innate immune sensors.

## 4 ADAR1 interactome

Beyond its intrinsic RNA-editing activity, ADAR1 functions within a network of protein–protein interactions that critically modulate its editing efficiency, substrate selection, and immunosuppressive roles. Given ADAR1's broad substrate promiscuity and capacity for extensive editing, such regulation is likely essential to focus editing on the most immunogenic endogenous dsRNAs while avoiding inappropriate or excessive modification. In addition, interaction partners may enable editing-independent functions of ADAR1 in RNA metabolism and immune regulation. Interactions reported to date fall into several broad classes: direct domain-specific contacts, RNA-mediated associations within larger ribonucleoprotein (RNP) assemblies, and proteins that indirectly compete for RNA substrates of ADAR1 or process ADAR1 substrates or products to modulate ADAR1.

### 4.1 Direct protein interactions organized by ADAR1 domain

Previous co-immunoprecipitation, biochemical, and cellular studies have identified several direct ADAR1-interacting proteins. For example, ADAR1 was shown to bind Dicer through its RBD2, engaging Dicer's DUF283 and DEAD-box helicase domains in an RNA-independent manner (Ota et al., 2013). Gel-filtration analyses further indicate that ADAR1 and Dicer form a 1:1 heterodimer. Functionally, ADAR1 enhances Dicer-mediated pre-miRNA processing, promotes miRNA transfer, and strengthens RNA-induced gene silencing (Kawahara et al., 2007; Ota et al., 2013), but Dicer instead suppresses ADAR1 editing of miRNAs, likely by disrupting ADAR1 dimerization and/or occluding shared RNA substrates (Ota et al., 2013). ADAR1 may also directly modulate innate immune signaling with protein-protein interactions. Sinigaglia et al. showed that ADAR1's RBD3 interacts with PKR's kinase domain to prevent PKR activation, and the interaction persists after RNase treatment (Sinigaglia et al., 2024). Mutations that impair RBD3–PKR binding abolish PKR suppression. Similarly, ADAR1 RBD3 was reported to associate with the DHX9 helicase (Hong et al., 2018). DHX9 depletion selectively reduces editing at ADAR1-preferred sites (Hong et al., 2018). Beyond dsRNA editing, ADAR1 and DHX9 were shown to cooperate in maintaining R-loop homeostasis by promoting R-loop resolution (Cui et al., 2022).

### 4.2 RNA-mediated protein assemblies

Beyond its well-defined binding partners, ADAR1 is frequently detected within larger RNP assemblies for context dependent RNA editing and editing-independent functions. A prominent example is FMRP and FXR1P, which are disease-causing genes for Fragile X Mental Retardation**.** FMRP and FXR1P were reported to associate with ADAR1 in an RNA-independent manner, yet regulate editing largely through RNA-dependent recruitment or competition (Tran et al., 2019). FMRP promotes editing at selected sites by facilitating ADAR1 access without altering RNA structure, whereas FXR1P inhibits editing across many hypereditable loci by possibly competing for overlapping dsRNA regions (Tran et al., 2019). Similarly, NF90 is a dsRNA-binding protein that regulates mRNA stability, translation, and miRNA biogenesis (Grasso et al., 2020). ADAR1 co-immunoprecipitates with NF90/NF110 in an RNA-dependent manner and enhances NF90-mediated gene expression (Nie et al., 2005). Emerging evidence suggests that ADAR1 also participates in RNA processing independently of its deaminase activity. Sagredo et al. identified >400 ADAR1-associated proteins enriched for spliceosomal factors and showed that catalytically inactive or RNA-binding–deficient mutants still alter splicing (Sagredo et al., 2025). These findings indicate that ADAR1 may influence gene expression through structural or allosteric modulation of RNA-processing complexes, functioning as a broader regulator of RNA metabolism rather than solely as an adenosine deaminase.

### 4.3 Indirect protein partners of ADAR1

RNA-binding proteins can regulate ADAR1 by competing for shared RNA substrates or processing edited RNAs. ADAR3, a catalytically inactive paralog, competes with ADAR1 for duplex RNA and suppresses ADAR1 editing (Karki et al., 2021; Raghava Kurup et al., 2022). Co-IP studies failed to detect stable ADAR3–ADAR1 interaction, supporting a model of inhibition through RNA competition, though AlphaFold2-predicted heterodimerization remains to be experimentally evaluated (Fisher and Beal, 2024). Competition for specialized RNA conformations may also influence ADAR1 function. ADAR1 and ZBP1 are the only two human proteins with ZBDs for Z-RNA binding and likely compete for endogenous Z-RNA substrates for immune regulation (Karki et al., 2021). Moreover, Tudor-SN (TSN) directly binds and degrades inosine-containing duplexes, especially IU-rich hyperedited RNA generated by ADAR1 (Weissbach and Scadden, 2012). Hyperedited RNAs accumulate in stress granules and recruit TSN; ADAR1 p150 and TSN colocalize specifically under dsRNA stress (Weissbach and Scadden, 2012). Co-IP confirms ADAR1–TSN association, but current evidence suggests that this reflects co-assembly within SGs rather than a well-defined protein–protein interface (Gutierrez-Beltran et al., 2021; Weissbach and Scadden, 2012). Similarly, Endonuclease V selectively cleaves inosine-containing duplexes at I–U mismatches, acting in turnover of edited RNA rather than as a direct ADAR1 regulator (Morita et al., 2013).

## 5 Conclusion and perspectives

ADAR1 maintains innate immune homeostasis through an integrated system that combines a permissive, multidomain architecture with a heterogeneous endogenous dsRNA substrate landscape, further shaped by context-dependent regulation from interacting proteins. Recent structural and mechanistic studies have provided important insights into the architecture and dynamics of ADAR1. Combined with decades of work on ADAR1 and ADAR2, these advances reveal how the deaminase domain recognizes and engages dsRNA substrates, clarify potential roles of auxiliary domains, and offer clues to the basis of substrate selectivity. These molecular insights can guide the rational design of therapeutic guide RNAs by informing local sequence and structural features that enhance ADAR1 engagement and editing efficiency while minimizing bystander or off-target editing. They may also inform the design and optimization of chemical modifications on guide RNA to improve their delivery and stability. Despite this progress, key mechanistic questions remain unresolved. The kinetic steps governing RNA binding, target search, and deamination are still poorly defined, and the domain-specific contributions to recognizing and editing diverse endogenous RNAs for immune sensor suppression remain elusive. The mechanistic understanding will not only clarify the ADAR1 RNA editing landscape but also facilitate the design of therapeutic RNAs that more effectively recruit ADAR1 and maximize editing efficiency. In addition, proteins that directly bind or post-translationally modify ADAR1, thereby tuning its catalytic activity and immune regulatory functions, are only beginning to be characterized. Beyond its central roles in fundamental RNA biology and innate immune regulation, ADAR1 is frequently overexpressed in tumors to dampen antitumor immunity, and targeting ADAR1 is emerging as a promising strategy to enhance cancer immunotherapy (Ishizuka et al., 2019). Moreover, recent preclinical and clinical efforts using guide RNA–mediated ADAR1 editing offer exciting opportunities to treat genetic diseases without introducing permanent alterations to the heritable genome (Mullard, 2024). A more comprehensive mechanistic understanding of how ADAR1 edits RNA and modulates immune responses will be essential for guiding these therapeutic strategies and for unlocking the full potential of ADAR1.

## CRediT authorship contribution statement

**Xiangyu Deng:** Writing – review & editing, Writing – original draft, Visualization, Validation. **Mariam Elsharkawy:** Writing – review & editing, Writing – original draft, Visualization, Validation. **Yang Gao:** Writing – review & editing, Writing – original draft, Validation.

## Conflict of interest

The authors declare no competing interests.
